# Harnessing the Microbiomes of Suppressive Composts for Plant Protection: From Metagenomes to Beneficial Microorganisms and Reliable Diagnostics

**DOI:** 10.3389/fmicb.2020.01810

**Published:** 2020-07-30

**Authors:** Stefanie Lutz, Barbara Thuerig, Thomas Oberhaensli, Johanna Mayerhofer, Jacques G. Fuchs, Franco Widmer, Florian M. Freimoser, Christian H. Ahrens

**Affiliations:** ^1^Agroscope, Research Group Molecular Diagnostics, Genomics and Bioinformatics, Wädenswil, Switzerland; ^2^SIB Swiss Institute of Bioinformatics, Wädenswil, Switzerland; ^3^Research Institute of Organic Agriculture (FiBL), Department of Crop Sciences, Frick, Switzerland; ^4^Agroscope, Research Group Molecular Ecology, Zurich, Switzerland; ^5^Agroscope, Research Group Phytopathology and Zoology in Fruit and Vegetable Production, Wädenswil, Switzerland

**Keywords:** compost, organic farming, microbiome, biocontrol, metagenomics, genome assembly, strain collection, mechanism of action

## Abstract

Soil-borne diseases cause significant yield losses worldwide, are difficult to treat and often only limited options for disease management are available. It has long been known that compost amendments, which are routinely applied in organic and integrated farming as a part of good agricultural practice to close nutrient cycles, can convey a protective effect. Yet, the targeted use of composts against soil-borne diseases is hampered by the unpredictability of the efficacy. Several studies have identified and/or isolated beneficial microorganisms (i.e., bacteria, oomycetes, and fungi) from disease suppressive composts capable of suppressing pathogens (e.g., *Pythium* and *Fusarium*) in various crops (e.g., tomato, lettuce, and cucumber), and some of them have been developed into commercial products. Yet, there is growing evidence that synthetic or complex microbial consortia can be more effective in controlling diseases than single strains, but the underlying molecular mechanisms are poorly understood. Currently, a major bottleneck concerns the lack of functional assays to identify the most potent beneficial microorganisms and/or key microbial consortia from complex soil and compost microbiomes, which can harbor tens of thousands of species. This focused review describes microorganisms, which have been isolated from, amended to or found to be abundant in disease-suppressive composts and for which a beneficial effect has been documented. We point out opportunities to increasingly harness compost microbiomes for plant protection through an integrated systems approach that combines the power of functional assays to isolate biocontrol and plant growth promoting strains and further prioritize them, with functional genomics approaches that have been successfully applied in other fields of microbiome research. These include detailed metagenomics studies (i.e., amplicon and shotgun sequencing) to achieve a better understanding of the complex system compost and to identify members of taxa enriched in suppressive composts. Whole-genome sequencing and complete assembly of key isolates and their subsequent functional profiling can elucidate the mechanisms of action of biocontrol strains. Integrating the benefits of these approaches will bring the long-term goals of employing microorganisms for a sustainable control of plant pathogens and developing reliable diagnostic assays to assess the suppressiveness of composts within reach.

## Introduction

Soil-borne plant pathogens including fungi, oomycetes, bacteria, and viruses, as well as parasitic nematodes, can cause severe yield losses. Soil-borne diseases such as pre- and post-emergence damping-off, root, stem collar and crown rots, and vascular wilting can be found in many crops and are primarily caused by members of the oomycetes (e.g., *Pythium* sp. and *Phytophthora* spp.) and fungi (e.g., *Rhizoctonia solani*, *Sclerotium* spp., *Fusarium* sp., and *Verticillium* sp.), but also by bacteria (e.g., *Ralstonia solanacearum*, *Pectobacterium*, and *carotovorum*), viruses (e.g., beet necrotic yellow vein virus, soil-borne wheat mosaic virus, and peanut clump virus), and nematodes (e.g., *Meloidogyne* sp.) (Koike et al., 2003; Agrios, 2005; Noble and Coventry, 2005; Fry and Grünwald, 2010; Andika et al., 2016). Pathogens may survive in soil for many years as dormant resting stages (i.e., spores). Chemical fungicide treatments are often not effective enough against soil-borne diseases (Reddy, 2016; You et al., 2020) and are met by increasing levels of criticism and public concerns about negative effects, highlighting the urgency to search for viable alternatives. A combined approach to avoid severe crop yield losses due to soil-borne pathogens includes breeding and selection of appropriate crop varieties, crop rotation, soil drainage, avoidance of soil compaction, appropriate sowing dates, and application of organic amendments such as composts (Abawi and Widmer, 2000; Bailey and Lazarovits, 2003; Ghorbani et al., 2009; Reddy, 2016).

In this review we focus on compost, a valuable recycling product that is widely used in agriculture, viticulture, horticulture as well as private gardening, as an integrated part of a good agricultural practice. Composts contain significant amounts of nutrients including phosphorus, magnesium, potassium, and calcium. Furthermore, compost amendments improve organic matter content, soil structure, water holding capacity, microbial biomass, and activity (Eden et al., 2017). In addition, they can suppress soil-borne plant diseases, which, if not defeated, often result in serious yield losses. Composts can also contribute to improved resistance of plants to foliar diseases (Zhang et al., 1998; Vallad et al., 2003).

The disease-suppressive potential of composts against a wide spectrum of pathogens has been known for many decades and has been summarized by several authors (Bailey and Lazarovits, 2003; Noble and Coventry, 2005; Bonanomi et al., 2010; Noble, 2011), with some reviews focusing on the role of microorganisms in disease suppression (Fuchs, 2009; Hadar and Papadopoulou, 2012; Mehta et al., 2014). For instance, in a meta-analysis on publications for the time period from the 1970s until 2006, Bonanomi et al. (2007) found a disease-suppressive effect of composts in more than 50% out of 1,016 case studies, while disease-promoting effects were rather rare (below 12% of the case studies). Similarly, reviewing reports of 79 container and 54 field experiments, Noble (2011) reports success rates of compost of 74% in container and 83% in field experiments, whereas a disease promoting effect was only found in 8 or 2% of the experiments, respectively.

Despite the overwhelming potential of composts to reduce soil-borne diseases in general, predictability of the success of the application of a certain compost against a particular pathogen is limited. Comparing the suppressiveness of 18 composts in seven different plant–pathogen systems, Termorshuizen et al. (2006) found overall success rates of 54%, but variability between pathogens was high, with success rates ranging from 6% for *Phytophthora cinnamomi* to 71% for *Phytophthora nicotianae*. Similarly, the review by Bonanomi et al. (2007) on 1,016 case studies revealed that the success rates of compost applications varied substantially between plant–pathogen systems, with success rates between 32% (*Rhizoctonia* spp.) and 74% (*Fusarium* sp.). To optimally exploit the disease-suppressive potential of composts in practice, predictability is highly desirable, but is so far hampered by the complexity of compost microbiota, which can contain thousands of different species or strains.

Compost is an organic material resulting from the mostly aerobic decomposition of organic matter by microorganisms. The composition of the starting material can be highly diverse, including different kinds of manure, wood, green waste, food waste, digestate (i.e., the remains from anaerobic decomposition in biogas production), or waste from specialized industries (e.g., olive mills and paper mills) (Fuchs et al., 2006). Professional producers of composts often add small amounts of former compost lots at the beginning of the composting process to inoculate microorganisms that promote the composting process. The composition of the microbial community highly depends on the initial compost material and undergoes a number of substantial alterations during the different phases (Mehta et al., 2014). A first mesophilic phase with temperatures of 25–40°C is generally followed by a thermophilic phase (40–70°C) during which thermophilic bacteria (e.g., *Bacillus* sp. and *Thermus* sp.) predominate. This phase is very important to destroy potentially harmful organisms, including human and plant pathogens as well as weed seeds (Bollen et al., 1989; Johansen et al., 2013). In the following cooling phase (second mesophilic phase), mesophilic organisms recolonize the substrate, either from protected niches such as edges of compost piles, from spores, or by external re-inoculation. In this phase, degraders of cellulose, including bacteria (e.g., *Cellulomonas* sp., *Clostridium* sp., and *Nocardia* sp.) as well as fungi (e.g., *Aspergillus* sp., *Fusarium* sp., and *Paecilomyces* sp.), become important (Insam and de Bertoldi, 2007). In the maturation or curing phase, the microbial community is again completely altered. Often fungi become predominant over bacteria due to higher competitiveness in conditions of poor nutrient availability. During storage, the microbial composition undergoes further alterations, while physico-chemical parameters remain relatively stable (Danon et al., 2008). Thus, the maturation stage and the storage of a mature compost are the main determinants for the microbial composition of the final compost product (Insam and de Bertoldi, 2007). However, the microbial composition of composts can also be influenced by the site of composting (e.g., composting facilities and field edges), the humidity management, initial C/N ratios, as well as turning techniques and intensities, which are all affecting the temperatures reached during the composting process (Eiland et al., 2001; Tiquia, 2005; Wang et al., 2015). Since many factors can affect compost quality, checking physical, chemical and biological properties of composts before application is essential, and quality guidelines have been set up in many countries by the government and the composting industry (e.g., Abächerli et al., 2010).

In some cases, abiotic factors have been shown to cause disease suppression by composts (e.g., high levels of ammonia in non-mature composts, high pH, fungitoxic compounds like hydroxyl-oleic acids, siderophores, and salts) (de Bertoldi, 2009). Yet, many studies have demonstrated a loss of disease suppressiveness in sterilized composts (Gorodecki and Hadar, 1990; Hoitink et al., 1997; Cotxarrera et al., 2002; Reuveni et al., 2002; Tilston et al., 2005), which indicates a crucial role of the living microorganisms in disease suppressiveness of composts. The ability of composts to suppress pathogens has been attributed either to the total microbial quantity and diversity (general disease suppression) or to individual organisms (specific disease suppression) (Berendsen et al., 2012).

It has been demonstrated that a change in the microbial community structure of soils occurs after the amendment with compost (Noble and Coventry, 2005) either by importing new microorganisms and/or by influencing the native microbial community. Several microorganisms isolated from or identified in compost have the potential to suppress soil-borne diseases and are summarized in the present review. Yet, attempts to generally predict the suppressiveness of composts against specific pathogens based on the presence/absence of known biocontrol strains and/or on other biotic (e.g., enzyme activity and microbial respiration) or abiotic markers (e.g., carbon, ammonia, and nitrate) have not been successful so far (de Bertoldi, 2009; Hadar and Papadopoulou, 2012). Furthermore, farmers often report superior disease-suppressive effects of composts compared to application of single strains (Jacques Fuchs, personal communication), even though the efficacy of biocontrol strains isolated from composts has been demonstrated under field conditions in several cases (Cao et al., 2011; Xue et al., 2015). There is growing evidence that an optimal disease suppression by composts might rather result from a consortium of microorganisms than from individual, specific strains. This is in accordance with many recent studies showing superior disease suppression of microbial consortia compared to individual strains (Berendsen et al., 2018).

Recent advances in next-generation sequencing (NGS) technology, such as amplicon and shotgun sequencing, have allowed characterizing the composition of compost microbiomes in great detail (Blaya et al., 2016). NGS technologies thus hold great potential to enable researchers to identify microorganisms and communities with a potential disease-suppressive effect. In addition, the *de novo* assembly of complete genomes of isolates that show a suppressive effect, offer unprecedented opportunities to move beyond studying the composition of compost microbiomes toward obtaining a mechanistic understanding of the beneficial effects that individual strains and consortia of strains can exert, and to elucidate direct causal effects (i.e., specific genes or pathways involved in suppression). The great potential of this approach has been shown for both strains isolated from natural soils that exert suppressive effects against pathogens (Mendes et al., 2011; De Vrieze et al., 2015; De Vrieze et al., 2020), as well as for strains isolated from the phyllosphere (Gore-Lloyd et al., 2019).

Here, we summarize the current knowledge on disease-suppressive isolates from compost for the biocontrol of plant pathogens and highlight the potential of an integrated systems approach combining experimental and NGS techniques to identify key microorganisms and microbial communities involved in suppressiveness of compost against plant diseases.

## Suppressive Microorganisms Associated With Compost

Several microorganisms isolated from or identified in compost have the potential to suppress soil-borne diseases. So far, these comprise taxa that are easily culturable under laboratory conditions (Schloss and Handelsman, 2004), which allows a later use as biocontrol agents or biofertilizers. Several organisms also occurring in composts (e.g., *Bacillus amyloliquefaciens*, *Streptomyces* sp., *Bacillus subtilis*, and *Trichoderma harzianum*) have been developed into commercial products (Bundesamt für Landwirtschaft [BLW], 2020; Speiser et al., 2020).

Several modes of action conveying a disease-suppressive effect have been described (Freimoser et al., 2019). Direct biological mechanisms include the overall increase in biomass and activity and therefore competition for nutrients and space among the different communities (“competition”) (Hadar and Mandelbaum, 1986; Mandelbaum, 1990; Diánez et al., 2005; Bonanomi et al., 2010; Bonilla et al., 2012), the direct attack of the pathogen through the production of secondary antimicrobial metabolites (“antagonism”) (Mehta et al., 2014), as well as the secretion of chitinases, glucanases, and proteases (“hyperparasitism”) (Nelson, 1983; Kwok et al., 1987; de Bertoldi, 2009). More indirect mechanisms are the activation of disease resistance genes in plants (i.e., induced systemic resistance, ISR) (Zhang, 1996; Zhang et al., 1998; Krause et al., 2003; Aldahmani et al., 2005; Horst et al., 2005; Kavroulakis et al., 2005; Ntougias et al., 2008), as well as an overall improvement of plant nutrition and vigor leading to enhanced disease resistance (Hameeda et al., 2006; Zhao et al., 2017).

So far, few studies have employed cultivation-independent approaches to characterize the microbiota of disease-suppressive composts using the 16S, 18S, and ITS rRNA markers (Yu et al., 2015; Blaya et al., 2016; Vida et al., 2016; Ros et al., 2017, 2020; Corato and De Corato, 2019; Corato et al., 2019; Scotti et al., 2020). The combined outcome of these studies shows that the taxa identified as highly abundant (see Tables 1, 2, method “abundant”) greatly vary depending on the studied plant–pathogen systems and the starting material of the compost. For instance, Blaya et al. (2016) found a higher abundance of Bacteriodetes, alpha- and gamma-Proteobacteria and Chloroflexi, as well as non-pathogenic *Fusarium* and *Zopfiella* in composts that were most suppressive against *Phytophthora nicotianae* in pepper. In contrast, Acidobacteria Gp14, Actinobacteria, and Cystobasidiomycetes were more abundant in composts with strong suppression of *Pythium* wilt disease in cucumber (Yu et al., 2015).

**TABLE 1:** List of identified beneficial bacteria either isolated from, amended to or abundant in suppressive compost, their respective plant–pathogen system(s) and (if known) the proposed mechanism of action conveying the suppressive effect.

**Beneficial organism**	**Method**	**Pathogen**	**Plant**	**Mechanism**	**References**
*Aeromonas media*	Isolated and amended	*Pythium ultimum*	Cress	Unknown	Oberhaensli et al. (2017)
Acidobacteria Gp14	Abundant	*Pythium* sp.	Cucumber	Unknown	Yu et al. (2015)
*Acinetobacter* sp.	Isolated	*Phytophthora capsici*, *P. citricola*, *P. palmivora*, *P. cinnamomi*	–	Unknown	Syed-Ab-Rahman et al. (2018)
*Bacillus amyloliquefaciens*	Isolated	*Fusarium oxysporum* f. sp. *cucumerinum* (FOC)	Cucumber	Possibly protease, cellulase and 1-aminocyclopropane-1-carboxylate (ACC) deaminase activity	Du et al. (2017)
	Isolated	*Phytophthora capsici*, *P. citricola*, *P. palmivora*, *P. cinnamomi*	–	Unknown	Syed-Ab-Rahman et al. (2018)
JDF35	Amended	*Fusarium oxysporum*	Watermelon	Promotion of plant growth (possibly via tryptophan-dependent synthesis of auxins and extracellular phytase activity and increased availability of N, P and K in the soil)	Zhao et al. (2017)
NJN-6	Amended	*Fusarium oxysporum*	Banana	Unknown	Xue et al. (2015)
*Bacillus licheniformis*	Isolated	*Fusarium oxysporum* f. sp. *cucumerinum* (FOC)	Cucumber	Possibly protease, cellulase and 1-aminocyclopropane-1-carboxylate (ACC) deaminase activity	Du et al. (2017)
	Isolated	*Verticillium dahliae, Fusarium oxysporum f.* sp., *Lycopersici* (FORL)	Tomato	Production of diffusible secondary metabolites (more than volatile organic compounds), indole-3-acetic acid (IAA) production, ACC deaminase activity	Tsolakidou et al. (2019)
*Bacillus subtilis* SQR 9	Isolated	*Verticillium dahliae, Fusarium oxysporum f.* sp., *Lycopersici* (FORL)	Tomato	Production of diffusible secondary metabolites (more than volatile organic compounds), indole-3-acetic acid (IAA) production, ACC deaminase activity	Tsolakidou et al. (2019)
*Bacillus tequilensis* CE4	Isolated	*Ganoderma boninense*	–	Unknown	Chin et al. (2017)
*Bacillus velezensis*	Isolated	*Phytophthora capsici*, *P. citricola*, *P. palmivora*, *P. cinnamomi*	–	Unknown	Syed-Ab-Rahman et al. (2018)
*Burkholderia* spp.	Abundant	*Rosellinia necatrix*	Avocado	Unknown	Vida et al. (2016)
*Chryseobacterium* sp.	Isolated	*Verticillium dahliae, Fusarium oxysporum* f. sp., *Lycopersici* (FORL)	Tomato	Production of diffusible secondary metabolites (more than volatile organic compounds), indole-3-acetic acid (IAA) production, ACC deaminase activity	Tsolakidou et al. (2019)
*Enterobacter* spp.	Abundant	*Pythium* sp.	Cucumber	Unknown	Chen et al. (2012)
	Isolated	*Verticillium dahliae, Fusarium oxysporum* f. sp., *Lycopersici* (FORL)	Tomato	Production of diffusible secondary metabolites (more than volatile organic compounds), indole-3-acetic acid (IAA) production, ACC deaminase activity	Tsolakidou et al. (2019)
*Enterobacter cloacae* subsp. *dissolvens* B3	Isolated	*Ganoderma boninense*	–	Unknown	Chin et al. (2017)
*Flavobacterium balustinum*	Amended	*Rhizoctonia*	Bark	Unknown	Kwok et al. (1987)
*Lechevalieria* sp.	Isolated	*Fusarium oxysporum* f. sp. *melonis, Phytophthora cinnamomi, Pythium debaryanum, Sclerotinia sclerotiorum, Thanatephorus cucumeris, Agrobacterium tumefaciens*	–	Possibly synthesis of antibiotic compounds	Cuesta et al. (2012)
*Nocardiopsis* spp.	Abundant	*Rhizocotnia solani, Sclerotinia minor*	Cress	Unknown	Scotti et al. (2020)
*Ochrobactrum* sp.	Isolated	*Verticillium dahliae, Fusarium oxysporum* f. sp. *Lycopersici* (FORL)	Tomato	Production of diffusible secondary metabolites (more than volatile organic compounds), indole-3-acetic acid (IAA) production, ACC deaminase activity	Tsolakidou et al. (2019)
*Paenibacillus polymyxa*	Isolated	*Fusarium oxysporum* f. sp. *cucumerinum* (FOC)	Cucumber	Possibly protease, cellulase and 1-aminocyclopropane-1-carboxylate (ACC) deaminase activity	Du et al. (2017)
*Pseudomonas* spp.	Abundant	*Pythium* sp.	Cucumber	Unknown	Chen et al. (2012)
	Abundant	*Rhizocotnia solani, Sclerotinia minor*	Cress	Unknown	Scotti et al. (2020)
	Abundant	*Rosellinia necatrix*	Avocado	Unknown	Vida et al. (2016)
*Serratia marcescens*	Isolated	*Fusarium oxysporum* f. sp. *radicis-lycopersici* (FORL)	Tomato	Unknown	Kavroulakis et al. (2010)
*Stenotrophomonas maltophilia*	Isolated	*Verticillium dahliae, Fusarium oxysporum* f. sp. *Lycopersici* (FORL)	Tomato	Production of diffusible secondary metabolites (more than volatile organic compounds), indole-3-acetic acid (IAA) production, ACC deaminase activity	Tsolakidou et al. (2019)
*Streptomyces albogriseolus*	Isolated	*Fusarium oxysporum* f. sp. *melonis, Phytophthora cinnamomi, Pythium debaryanum, Sclerotinia sclerotiorum, Thanatephorus cucumeris, Agrobacterium tumefaciens*	–	Possibly synthesis of antibiotic compounds	Cuesta et al. (2012)
*Streptomyces aureoverticillatus*	Isolated	*Fusarium oxysporum* f. sp. *melonis, Phytophthora cinnamomi, Pythium debaryanum, Sclerotinia sclerotiorum, Thanatephorus cucumeris, Agrobacterium tumefaciens*	–	Possibly synthesis of antibiotic compounds	Cuesta et al. (2012)
*Streptomyces coeruleorubidus*	Isolated	*Fusarium oxysporum* f. sp. *melonis, Phytophthora cinnamomi, Pythium debaryanum, Sclerotinia sclerotiorum, Thanatephorus cucumeris, Agrobacterium tumefaciens*	–	Possibly synthesis of antibiotic compounds	Cuesta et al. (2012)
*Streptomyces griseoruber*	Isolated	*Fusarium oxysporum* f. sp. *melonis, Phytophthora cinnamomi, Pythium debaryanum, Sclerotinia sclerotiorum, Thanatephorus cucumeris, Agrobacterium tumefaciens*	–	Possibly synthesis of antibiotic compounds	Cuesta et al. (2012)
*Streptomyces lusitanus*	Isolated	*Fusarium oxysporum* f. sp. *melonis, Phytophthora cinnamomi, Pythium debaryanum, Sclerotinia sclerotiorum, Thanatephorus cucumeris, Agrobacterium tumefaciens*	–	Possibly synthesis of antibiotic compounds	Cuesta et al. (2012)
*Streptomyces variegatus*	Isolated	*Fusarium oxysporum* f. sp. *melonis, Phytophthora cinnamomi, Pythium debaryanum, Sclerotinia sclerotiorum, Thanatephorus cucumeris, Agrobacterium tumefaciens*	–	Possibly synthesis of antibiotic compounds	Cuesta et al. (2012)

*There are large variations between taxa identified in isolation (method “isolated”) and NGS (method “abundant”) studies, which can be attributed in parts to the fact that NGS approaches also sample unculturable microorganisms.*

**TABLE 2:** List of identified beneficial fungi and oomycetes either isolated from, amended to or abundant in suppressive compost, their respective plant–pathogen system(s) and (if known) the proposed mechanism of action conveying the suppressive effect.

**Beneficial organism**	**Method**	**Pathogen/Parasite**	**Plant**	**Mechanism**	**References**
*Coniothyrium minitans*	Amended	*Sclerotinia minor*	Lettuce	Possibly nutrient/space competition	Rabeendran et al. (2006)
Cystobasidiomycetes	Abundant	*Pythium* sp.	Cucumber	Unknown	Yu et al. (2015)
Dothideomycetes	Abundant	*Rosellinia necatrix*	Avocado	Unknown	Vida et al. (2016)
Non-pathogenic *Fusarium oxysporum*	Amended	*Rhizoctonia solani*	Carnation	Unknown	Postma et al. (2003)
	Abundant	*Phythohthora nicotinanae*	Pepper	Unknown	Blaya et al. (2016)
Non-pathogenic *Pythium sp. RB II*	Isolated and amended	*Pythium mamillatum, Pythium pyrilobum, Pythium irregular, Phytophthora cinnamomi*	Rooibos	Possibly nutrient competition	Bahramisharif et al. (2013)
Non-pathogenic *Pythium acanthicum*	Isolated and amended	*Pythium mamillatum, Pythium pyrilobum, Pythium irregular, Phytophthora cinnamomi*	Rooibos	Possibly nutrient competition	Bahramisharif et al. (2013)
Non-pathogenic *Pythium cederbergense*	Isolated and amended	*Pythium mamillatum, Pythium pyrilobum, Pythium irregular, Phytophthora cinnamomi*	Rooibos	Possibly nutrient competition	Bahramisharif et al. (2013)
*Trichoderma asperellum*	Amended	*Phytophthora nicotianae*	Pepper	Mycoparasitism, antibiosis, nutrient/space competition	Ros et al. (2017)
	Isolated and amended	*Fusarium oxysporum*	Tomato	Unknown	Cotxarrera et al. (2002)
T34	Amended	*Fusarium oxysporum*	Carnation	Possibly induction of plant disease resistance, increased root system and available surface area, facilitated water uptake	Sant et al. (2010)
T34	Amended	*Rhizoctonia solani*	Cucumber	Chitinase activity	Trillas et al. (2006)
*Trichoderma harzianum*	Amended	*Phytophthora nicotianae*	Pepper	Mycoparasitism, antibiosis, nutrient/space competition	Ros et al. (2017)
	Amended	*Rhizoctonia solani*	Radish	Unknown	Nelson (1983)
*Verticillium biguttatum*	Amended	*Rhizoctonia solani*	Sugar beet, Potato	Unknown	Postma et al. (2003)
*Zopfiella*	Abundant	*Phythohthora nicotinanae*	Pepper	Unknown	Blaya et al. (2016)

*There are large variations between taxa identified in isolation (method “isolated”) and NGS (method “abundant”) studies, which can be attributed in parts to the fact that NGS approaches also sample unculturable microorganisms.*

Due to PCR amplification biases in amplicon-based studies (Sambo et al., 2018), a considerable fraction of organisms contributing to diseases suppression of compost is likely hitherto unexplored. Yet, at the moment, these marker-based studies are the only feasible high-throughput method for a large number of samples. Very few studies have employed a shotgun approach. For instance, Antunes et al. (2016) have described the microbial community structure during a composting process using shotgun as well as amplicon-based metagenomics. They found that the two data sets were mostly in accordance on higher taxonomic levels, whereas the largest discrepancies could be found on the species level. More compost microbiome studies are needed to get a better understanding of these complex systems, to elucidate which bacterial or fungal species correlate with disease suppression, and to increase the number of functionally relevant isolates as starting point for the design of robust consortia for biocontrol applications.

### Bacteria

Bacterial communities in compost are usually characterized by a high abundance of the phyla Proteobacteria, Firmicutes, Bacteroidetes, and Actinobacteria (Neher et al., 2013). These phyla are commonly present in composts (and soils) regardless of their maturity and chemical and physical properties (Yu et al., 2015).

Within the Gammaproteobacteria, several *Pseudomonas* spp. and *Enterobacter* spp. strains are known to suppress *Pythium* damping-off (Chen et al., 2012) (Table 1). *Pseudomonas* is one of the most diverse (phylogenetically and functionally) Gram-negative bacterial genera with the largest number of known species (Gomila et al., 2015). It is ubiquitous in a wide range of habitats and exhibits a high degree of physiological and genetic adaptability (Mulet et al., 2012). For instance, *Pseudomonas* strains were abundant in a compost with a disease-suppressive effect toward *Pythium* in cucumber (Chen et al., 2012). *Enterobacter cloacae* has been isolated from compost and showed promising anti-*Ganoderma* activity (Chin et al., 2017). Besides *Pseudomonas* and *Enterobacter*, other genera of the Gammaproteobacteria can also contribute to disease-suppressive traits of composts. For instance, *Aeromonas media* strains were preferentially isolated from the rhizoplane of plants grown in substrates amended with suppressive compost (Oberhaensli et al., 2017). *A. media* improved suppressiveness of compost against *P. ultimum* in cress (*Lepidium sativum* L.) potting experiments. The largest effect was achieved when the bacterial strains were added to compost with a low suppressiveness, which increased the suppressiveness to the level of highly suppressive compost (Oberhaensli et al., 2017).

Within the phylum Firmicutes, *Bacillus* (e.g., *B. subtilis*) and related genera have demonstrated a beneficial effect on the disease suppression activity of compost (Nakasaki et al., 1998; Cao et al., 2011; Antoniou et al., 2017) (Table 1). *Bacillus amyloliquefaciens* amended to compost increased the suppression of *Fusarium* wilt disease in watermelon (Zhao et al., 2017), banana (Xue et al., 2015), as well as in cucumber (Du et al., 2017). Other *Bacillus* strains isolated from disease-suppressive composts include *B. licheniformis* (Du et al., 2017), *B. subtilis* (Tsolakidou et al., 2019), *B. tequilensis* (Chin et al., 2017), and *B. velezensis* (Syed-Ab-Rahman et al., 2018) (Table 1).

Within the phylum Actinobacteria, several *Streptomyces* strains including *S. aureoverticillatus*, *S. coeruleorubidus*, *S. griseoruber*, *S. lusitanus*, and *S. variegatus* have been isolated from compost and showed antagonistic activity toward several phytopathogens (e.g., *Pythium*, *Fusarium*, and *Phytophthora*) (Cuesta et al., 2012).

Within the phylum Bacteroidetes, Sphingobacteria as well as Flavobacteria (e.g., *Flavobacterium balustinum*) have been associated with disease suppression (Kwok et al., 1987). Similarly, the higher abundance of the subgroup Gp14 of the phylum Acidobacteria in compost was associated with suppressiveness (Yu et al., 2015) (Table 1).

### Oomycetes and Fungi

Several beneficial oomycetes and fungi have been associated with suppressiveness of compost (Table 2). A higher abundance of Cystobasidiomycetes has been found in composts with a stronger disease-suppression ability compared to composts without or with a reduced ability (Yu et al., 2015). The fungal antagonist *Verticillium biguttatum* was isolated from compost and its addition to compost increased the suppressiveness in bioassays with *Rhizoctonia solani* and sugar beet or potato (Postma et al., 2003). Non-pathogenic *Pythium* taxa in combination with compost significantly reduced damping-off in rooibos caused by pathogenic *Pythium* (Bahramisharif et al., 2013). Addition of the biocontrol agent *Trichoderma asperellum* strain T-34 to compost reduced *Rhizoctonia* damping-off in cucumber plants (Trillas et al., 2006), as well as *Fusarium* wilt in tomato and carnation (Cotxarrera et al., 2002; Sant et al., 2010). Ros et al. (2017) have evaluated the effect of compost fortified with the biological control agent *Trichoderma* on pepper infected with *Phytophthora nicotianae*. They found that the amended *Trichoderma* likely conveys higher disease suppressiveness than the native compost microbiota.

## Current Challenges and Limitations

The application of compost and compost derived microorganisms for disease control entails various challenges and limitations (e.g., pathogen-specificity, unknown mechanisms, temporal variability of the same compost, large microbial diversity, and registration), which will be discussed in the following in detail.

In a large number of studies, a compost suppressive to one pathogen was ineffective or rarely even conducive to other pathogens, which suggests that suppressiveness is often pathogen-specific (Bonanomi et al., 2010) and that consortia of beneficial microorganisms may have to be specifically tailored to target different pathogens. Moreover, some pathogens appear to be very enigmatic. For instance, the pathogenicity of *R. solani* is still poorly understood despite the enormous number of studies that have aimed to find an effective control agent (total *n* = 670; *n* = 272 for composts) (Bonanomi et al., 2010). In general, most experiments have been conducted on fungal and oomycete pathogens so far (Noble, 2011).

The ability of certain composts to suppress diseases does not only vary across pathogens (Scheuerell et al., 2005; Termorshuizen et al., 2006), but even when using composts of very similar composition and at the same inclusion rate (volume compost/volume substrate) (Noble and Coventry, 2005). This variability impairs repeatability and reliability of experiments even if they are performed with highly similar batches of compost. This most likely can be attributed to the enormous variability of compost characteristics and the impossibility of obtaining composts with a standardized (i.e., identical) composition, as well as to changes in the microbial composition over time and during storage (van Rijn et al., 2007). The enormous diversity of the microbial compost communities poses big challenges for the identification of potential beneficial strains and consortia. Furthermore, it is challenging to identify direct, and in particular, indirect interactions among microbes jointly contributing to disease suppression (Chen et al., 2012). A large proportion of the compost microbes may not be involved in obvious binary interactions (e.g., pathogen – antagonist), but rather influence interactions between other organisms (i.e., tritagonists) (Freimoser et al., 2016). In order to develop individual strains or combinations of strains into biological control products, they first need to be isolated and for a large part of them this is not possible due to very specific or unknown requirements for successful cultivation (see section “An Integrated Strategy to Harness Compost Microbiomes for Plant Protection”). Furthermore, their registration as biopesticides (in the class of microbial pesticides) is required. The prerequisites for their registration can vary across countries and can be laborious, which further hampers their commercialization. Since these strains originate from compost, which has experienced elevated temperatures during the composting process, they often show a high heat resistance and can be very persistent (Jurado et al., 2014). As for any other biocontrol strain, questions related to biosafety and in particular the potential to colonize mammalian hosts have to be carefully studied. Despite the necessity to ensure biosafety, data requirements to register microbial biocontrol agents in the European Union are extensive, time-consuming and expensive (Köhl et al., 2019), thus detaining companies from submitting registration dossiers and resulting in very low numbers of newly approved microbial biocontrol organisms. In the case of synthetic microbial consortia, each single strain has to be approved by authorities, which even multiplies hurdles for registration. In other countries like the United States, more tailored registration procedures result in many more newly approved microbial biocontrol organisms (Köhl et al., 2019). Striving for similar procedures in the European Union by the biocontrol industry has not been successful so far (Anonymous, 2018; Köhl et al., 2019).

Considering the complex nature of composts, a multitude of factors likely contributes to suppressiveness. So far, no defined set of taxa has explained disease suppression and their composition will likely be context (host, pathogen, soil, or substrate) dependent. We consider an integrated systems approach very promising to overcome these challenges and outline such an approach in the following section. Possible important outcomes of such a systems approach may include identification and isolation of new biocontrol strains and their mode of action, development of microbial consortia with suppressiveness superior to single strains, development of diagnostic tools to allow a targeted application of composts against soil-borne diseases, and the development of strategies to selectively promote key microbial organisms in composts.

## An Integrated Strategy to Harness Compost Microbiomes for Plant Protection

**FIGURE 1:**
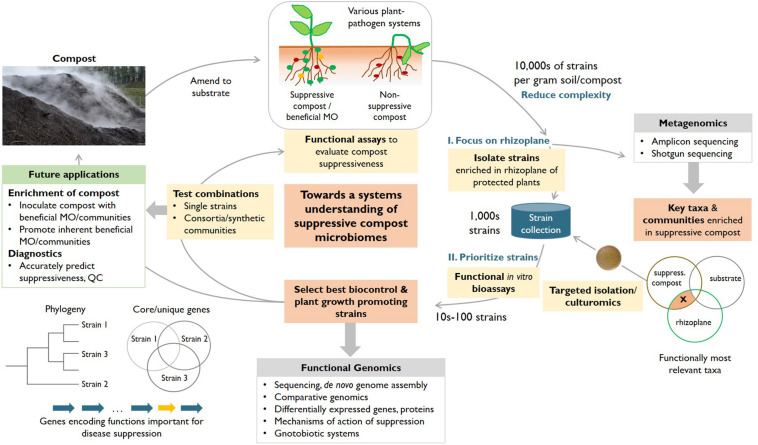
An integrated systems approach to enhance our understanding of the microorganisms responsible for the disease-suppressive effects of compost (red boxes) in various plant–pathogen systems. Our workflow integrates the combined power of experimental approaches (yellow boxes), metagenomics and functional genomics (gray boxes) of key isolates with the overall aim of identifying suppressive strains (or consortia) and their respective mechanisms of action. Critical elements include the reduction of complexity by focusing on strains enriched in the rhizoplane of plants treated with suppressive compost, robust functional assays to prioritize among a collection of rhizoplane isolates, feedback from metagenomics about conditions to potentially capture additional, so far unculturable relevant bacterial taxa and functional genomics to elucidate mechanisms of action (e.g., genes encoding functions important for disease suppression, orange arrow) and to create a basis for diagnostic tests that can accurately predict the suppressiveness of a compost. The collection of strains is useful to design improved consortia of microorganisms that can be amended to enhance the protective effect of composts (through biocontrol and plant growth promotion). MO, microorganisms.

In order to establish a basis to increasingly harness compost microbiomes for plant protection, we propose an integrated systems approach (Figure 1). This approach combines the power of experiments and functional assays (Figure 1, yellow boxes), and the value of a growing collection of isolated strains (Figure 1, blue container) with detailed metagenomics studies (i.e., amplicon and shotgun sequencing) to achieve a better understanding of the complex system compost and to identify members of taxa enriched in suppressive composts. Further, this approach includes whole-genome sequencing of potentially suppressive isolates and subsequent functional genomics studies (i.e., comparative genomics, (meta-)transcriptomics, and (meta)-proteomics), which can elucidate the mechanisms of action of biocontrol strains and represent a basis to amend composts with optimal strain combinations (biocontrol and plant growth promoting strains) and design specific diagnostic applications (Figure 1, green box).

The development of robust functional assays, which allow to accurately evaluate the suppressiveness of compost, is one of the most important tasks. Due to the above mentioned pathogen-specificity of suppressive composts, such assays should be developed for a broad range of plant–pathogen systems (Bahramisharif et al., 2013; Oberhaensli et al., 2017), and ideally should be miniaturized to allow sufficient throughput at manageable costs.

Once such robust assays are available, potentially beneficial microorganisms conveying the disease-suppressive effect can be isolated from suppressive composts (Figure 1). These strains would largely expand on the rather limited repertoire of suppressive microorganisms that have been described so far (see Tables 1, 2). In order to reduce the extremely high species diversity in composts and substrates/soil that can amount to tens of thousands of operational taxonomic units (OTUs) or exact sequence variants (ESVs), it is likely beneficial to target specific niches, for instance the rhizoplane (i.e., the region where the root surface is in contact with soil). Plants are able to shape their rhizoplane microbiome (Hu et al., 2018) and possibly attract microorganisms involved in disease suppression (Berendsen et al., 2012). Therefore, isolation experiments targeting the rhizoplane allows researchers to focus on the likely most relevant species (Figure 1, orange boxes). For instance, *A. media* was the dominant species in the rhizoplane of cress grown in a suppressive compost as compared to cress grown in non-suppressive composts. The addition of *A. media* to non-suppressive compost increased the suppressiveness against *P. ultimum* (Oberhaensli et al., 2017). Importantly, the creation of a collection of rhizoplane strains isolated from protected plants represents a critical first objective of the integrated systems approach and a highly valuable resource not only for subsequent functional genomics studies but also for an effort to develop optimized consortia of biocontrol and plant growth promoting strains.

Patterns of differential enrichment in the rhizoplane of protected plants (as observed for *A. media*) further contribute to the reduction of complexity and prioritization of the potentially most relevant microorganisms. Such an enrichment can also be identified using amplicon-based metagenomic diversity studies (16S, 18S, and ITS), which allow an assessment of the microbial community composition (including unculturable microorganisms) and dynamics for a large number of samples. For instance, Blaya et al. (2016) found a higher relative abundance of Ascomycota including the genera *Fusarium* and *Zopfiella* in highly suppressive composts against *Phytophthora* root rot. These microbiome studies can thus also help to further guide efforts to isolate key taxa and choosing the appropriate culturing techniques, as well as the design of synthetic communities (see below).

Controlled lab experiments with isolated and cultured strains (e.g., from the rhizoplane) are a crucial step toward a better understanding of their functional potential. However, the majority of microorganisms appear unculturable using conventional approaches, known as the “great plate count anomaly” (Staley and Konopka, 1985). Recent advances in culturomics, i.e., efforts to culture a larger number of isolates from microbiomes (Lagier et al., 2018; Sarhan et al., 2019), minimize the gap between the number of culturable species and the actual microbial richness and can even identify species that are not detected by sequencing-based approaches (Lagier et al., 2012). Culture conditions need to be adapted to the potentially beneficial taxa identified in the sequencing-based microbial community analysis in order to maximize the number of culturable and potentially new bacteria, fungi, and oomycetes. While such culturomics based approaches are challenging, they have greatly expanded the number of strains obtained from the human gut microbiome for example (Lagier et al., 2018). A similar approach also enabled the identification of the novel antibiotic teixobactin against Gram-positive bacteria (Ling et al., 2015), which was isolated from the previously uncultured soil bacterium *Eleftheria terrae* that was successfully grown in its natural habitat with a diffusion chamber (i.e., isolation chip) (Nichols et al., 2010). Individual strains are then characterized and prioritized according to their suppressiveness in functional assays (Hilber-Bodmer et al., 2017). Further, public strain collections may represent a valuable resource to explore the disease-suppressive potential of additional strains from the same species or genus. Eventually, the beneficial strains need to be tested *in vivo* (i.e., *in planta*), since several studies have shown that *in vitro* assays often fail to capture the complexity of a natural system including plant-associated phenotypes (Bulgarelli et al., 2013). Since disease suppression likely does not derive from a single strain, these experiments should explore the composition of various consortia to point toward a particularly potent microbial community for plant protection (De Vrieze et al., 2018; Tsolakidou et al., 2019). Consortia often result in better plant growth promotion or protection in comparison to single strains (Berendsen et al., 2018).

Whole-genome sequencing (using long reads NGS technologies such as Pacific Biosciences and Oxford Nanopore Technologies), complete *de novo* genome assembly, and genome annotation of the most promising candidates will be the basis for an in-depth elucidation of modes of action and provide important information for predicting metabolic features (Schmid et al., 2018). This approach represents an optimal basis for subsequent downstream genome mining and ‘omics analyses (Dunlap et al., 2013; Van Der Voort et al., 2015; Mullins et al., 2019). Based on these annotated genomes, genes potentially involved in the process of disease suppression, and thus, the respective mechanisms of action underlying a suppressive effect, can be identified. This can entail a genome mining effort to identify genes from gene families already known to play a role in biocontrol (these include volatiles, secretion systems and their effectors, proteases, chitinases, and glucanases). Alternatively, the gene inventories of beneficial strains and closely related non-beneficial strains can be compared using comparative genomics approaches, or even more informative, the genomes of suppressive strains versus the genomes of mutant strains that have lost the ability to suppress a pathogen. For instance, whole-genome sequencing of both wild-type and a pigmentless mutant strain of the antagonist *Metschnikowia pulcherrima* allowed to identify Snf2 as a regulator of antifungal activity (Gore-Lloyd et al., 2019). Thereby, links from the genotype to a phenotype can be suggested, as has been shown in systems for potential biocontrol strains and different host plant varieties (De Vrieze et al., 2020). An important approach to reduce the number of candidate genes provided from genome comparisons are transcriptomics studies. If carried out on relevant conditions (e.g., suppression versus no effect), they can help to identify the most relevant differentially expressed genes and allow to generate testable hypotheses about the mechanism of action involved. Ideally such experiments are carried out using gnotobiotic systems, which represent a controlled environment using sterilized substrate, and thus, confounding factors (e.g., other soil microbiome strains) are eliminated (Timmusk and Wagner, 1999). Such systems allow investigating the interactions between a certain pathogen, the infected plant and the suppressiveness of a beneficial strain. For instance, a well-studied system is the activated ISR in *Arabidopsis thaliana* caused by rhizosphere inhabiting strains of *Pseudomonas fluorescens* (Bakker et al., 2007). Furthermore, the design and testing of various synthetic communities in these gnotobiotic systems can help explain the observed protection of the plant against the respective pathogen. Further, traits of a consortium in comparison to single strains can be explored. A drawback of this method is that some suppressive features may only be active under specific environmental conditions. For instance, several microbial antagonists decrease their chitinase production in the presence of simple sugars (de la Cruz et al., 1993; Lorito, 1993; Gupta et al., 1995). Thus, in order to efficiently employ suppressive strains, the appropriate abiotic and biotic conditions need to be known.

Targeted functional (Dougherty et al., 2012; Yeh et al., 2013) or shotgun metagenomic approaches can further inform about potential community functions. For instance, new glycoside hydrolases, which play important roles in degradation of biomass, were discovered in a microbial compost community using shotgun metagenomics (Dougherty et al., 2012). Similarly, compost metagenomes can be mined for genes potentially involved in disease suppression based on homologous genes in databases. Genes with suppressive traits can then be used as markers to screen composts for their disease suppression potential.

In order to elucidate actual functional activity, meta-transcriptomics, in this case of the entire community instead of isolated strains, is a promising technology. It can significantly reduce the complexity by targeting the expressed genes rather than the entire genetic information that is contained in a metagenome. This has been successfully demonstrated in soil studies (Shrestha et al., 2009; Hultman et al., 2015) and in a time-series of a composting process (Antunes et al., 2016). A comparison of gene expression profiles of suppressive and non-suppressive composts could provide a better understanding of the underlying mechanisms of disease suppression. Proteomics is another promising technology for future compost studies since it provides more accurate data about the expression, modification and interaction status of the actual players that carry out most functions in cells, i.e., the proteins (Ahrens et al., 2010; Aebersold and Mann, 2016). In this context, meta-proteomic studies could further extend the functional data sets collected from compost microbiomes. Although such an approach still presents considerable challenges for highly complex microbiomes such as soils and composts, they have the potential to link microbial community compositions and functions (Wilmes and Bond, 2006). For instance, Liu et al. (2015) elucidated the main biodegradation pathways in a composting system and could link them to specific organisms. Similarly, proteins involved in disease suppression could be attributed to beneficial organisms in suppressive compost. Notably, a meta-proteomic analysis will largely benefit from the growing inventory of whole genome sequences from well-characterized entries of strain collections, as this will allow creating better protein search databases, for which the genomic sequence information is required. With complete genomes in hand, proteogenomics (Omasits et al., 2017) will be instrumental to identify as of yet missed biocontrol genes such as short lipopeptides (Van Der Voort et al., 2015) allowing to further extend the inventory of the genetic determinants of antagonism. Finally, the analysis of the metabolome represents an important aspect to monitor the ultimate response of an organism to its environment. Metabolites are conserved across taxa and can therefore be investigated even if no reference genomes are available. Thus, metabolomics represents another powerful tool for the identification of disease-suppressive mechanisms. For instance, Vinci et al. (2018) showed an enhanced phosphorus and nutrient uptake in maize leaves, and thus improved photosynthetic activity, when treated with *B. amyloliquefaciens* in combination with composts.

Ultimately, the combined information gained from the described approaches will help to develop specific diagnostic tests that can predict the suppressive potential of a given compost for individual plant–pathogen systems, which would further advance the large-scale application of composts to suppress soil-borne diseases. A combined approach including bioassays, metagenomics, strain collections and subsequent sequencing and functional genomics of key isolates is crucial. While metagenomic studies can find patterns in microbial community structures, and thus, can point toward potentially beneficial taxa, isolates with suppressive traits are indispensable for conducting controlled experiments and building up a collection of strains from various plant–pathogen systems. These compost strain collections form the basis to identify consortia of beneficial microorganisms that act synergistically to achieve an overall enhanced biocontrol effect.

In conclusion, the application of compost for disease suppression has a huge potential. However, so far the overall number of isolated strains with documented suppressive effects is rather low and the interactions between the beneficial microbiota, pathogens, and host plants, as well as the mechanisms of action are mostly unknown. A deeper mechanistic understanding will allow to develop tailored solutions for specific phytosanitary problems, for example, by testing composts for suitable microbial compositions or by targeted enrichment with microorganisms adapted for the respective application.

## Data Availability Statement

All relevant data is contained within the article.

## Author Contributions

SL wrote the manuscript with substantial input from BT (compost background), FF (mechanism of action), and CA (functional genomics). TO carried out the initial literature search and created the final figure with CA. JM, JF, FW, and TO provided critical feedback to improve the manuscript. All authors contributed to the figure, tables, and literature search. All authors contributed to the article and approved the submitted version.

## Conflict of Interest

The authors declare that the research was conducted in the absence of any commercial or financial relationships that could be construed as a potential conflict of interest.
